# Ultrasound-guided platelet-rich plasma injection for peripheral nerve injury: a retrospective exploratory study

**DOI:** 10.3389/ebm.2026.11091

**Published:** 2026-10-26

**Authors:** Juanjuan He, Li Jiang, Anna Tu, Na Li, Hongyan Zhu, Ying Lu, Xiaomei Wei

**Affiliations:** 1 Third Affiliated Hospital of Sun Yat-sen University, Guangzhou, China; 2 The Third Affiliated Hospital, Southern Medical University, Guangzhou, China; 3 Zhaoqing Hospital, Third Affiliated Hospital of Sun Yat-sen University, Zhaoqing, China

**Keywords:** functional outcomes, peripheral nerve injury, platelet-rich plasma, safety, ultrasound guidance

## Abstract

Although experimental studies have demonstrated that platelet-rich plasma (PRP) promotes peripheral nerve repair, its clinical efficacy in treating peripheral nerve injuries remains unclear. The present study aims to evaluate safety and functional outcomes of ultrasound-guided PRP injection for peripheral nerve injury. This retrospective study analyzed consecutive patients receiving ultrasound-guided PRP injections for peripheral nerve injuries between January 2021 and March 2025. Outcomes were assessed at 1, 3, and 6 months post-treatment, defined as: (1) a ≥2-point reduction in the Numerical Rating Scale (NRS) for sensory recovery, and (2) a ≥1-grade improvement in the Medical Research Council (MRC) scale for motor function. Patients were divided based on symptom duration into short-duration (<1 year) and long-duration (≥1 year) groups. All procedure-related complications were recorded. Among 48 treated nerves, PRP therapy resulted in gradual functional improvement and was well-tolerated. Sensory recovery rates remained stable (70.8% at 1 month to 68.8% at 6 months), while motor function improved gradually (from 50.0% to 69.4%). In an exploratory subgroup analysis, the short-duration group (n = 25) showed numerically higher rates than the long-duration group (n = 23) at 6 months (sensory: 80.0% vs. 56.5%, p = 0.08; motor: 85.0% vs. 50.0%, p = 0.034). However, after Bonferroni correction for multiple comparisons (corrected α = 0.0167), the motor improvement did not remain statistically significant. No severe adverse events occurred. The present study suggests that ultrasound-guided PRP injection appears safe and may be associated with functional improvement. The observed subgroup findings are hypothesis-generating and require confirmation in prospective randomized controlled trials.

## Impact statement

This study provides preliminary evidence that ultrasound-guided PRP injection is safe and may be associated with functional improvement in peripheral nerve injuries. Patients with shorter symptom duration showed numerically higher improvement rates, but this finding did not withstand correction for multiple comparisons. Given the retrospective, uncontrolled design, these results are hypothesis-generating and require confirmation in prospective randomized controlled trials.

## Introduction

Peripheral nerve injuries are commonly encountered in clinical practice, with compression and trauma being the primary causes [1]. In contrast, factors such as inflammation, ischemia, poisoning, and iatrogenic injuries play a lesser role in the development of these injuries [2]. The incidence rate of carpal tunnel syndrome (CTS), the most prevalent form of compressive nerve injury, varies between 2.7% and 5.8% in the general population [3]. Additionally, it has been reported that approximately 2.3% of patients sustaining extremity trauma experience peripheral nerve injury [4].

Peripheral nerve injuries are characterized by neuropathic pain, sensory disturbances, and motor dysfunction in the affected area. Due to the diversity of mechanisms and varying degrees of severity, a multitude of treatment approaches are currently available for managing these conditions [1]. For instance, in cases of compression injuries, initial treatment typically involves conservative measures such as rest, physical therapy, acupuncture, medications and hydrodissection treatment [5, 6]. If conservative treatment measures fail, surgical intervention becomes necessary, but the surgery may pose a risk of secondary nerve compression due to inflammation or scar tissue formation [7]. In cases of peripheral nerve injuries resulting from trauma, surgery is often inevitable. Yet, even after surgery, some patients may not fully regain their sensory and motor functions [8]. Consequently, additional therapeutic strategies are necessary to facilitate neurological recovery.

PRP, derived from autologous blood, features platelet concentrations 3 to 5 times higher than normal and encompasses a range of growth factors such as platelet-derived growth factor, transforming growth factor beta, and vascular endothelial growth factor. PRP therapy leverages the efficacy of these platelet growth factors in supporting the wound healing and repair cascade, specifically the inflammation, proliferation, and remodeling phases [9]. While PRP therapy is prevalent in the treatment of musculoskeletal diseases [10], its application in peripheral nerve injuries remains less common. However, experimental studies have demonstrated PRP’s potential to enhance myelin sheath and axon repair [11] and alleviate neuropathic pain [12], suggesting its promising use in peripheral nerve injury treatment. Currently, reports on PRP’s utilization in peripheral neuropathy are scarce, with limited evidence suggesting its safety and effectiveness in treating conditions like CTS and diabetic peripheral neuropathy.

Crucially, the duration of denervation significantly impacts regenerative potential: chronic injuries exhibit diminished Schwann cell activity, axonal growth cone collapse, and irreversible muscle atrophy, leading to substantially poorer functional outcomes compared to acute/subacute cases. This study aims to retrospectively analyze the outcomes of patients with peripheral nerve injuries who have undergone PRP treatment in our department over recent years, providing preliminary insights into the safety and potential efficacy of PRP therapy for managing such injuries. In an exploratory analysis, we also compared outcomes between patients with shorter versus longer symptom duration to investigate whether disease duration may influence the therapeutic effects of PRP, recognizing that any such findings would be hypothesis-generating and require confirmation in future studies.

## Materials and methods

### Study design and population

The study protocol was approved by the Ethics Committee of the Third Affiliated Hospital of Sun Yat-sen University (approval No.II2024-022-01). Patients with peripheral nerve injury who have received PRP therapy in the rehabilitation department of Third Affiliated Hospital, Sun Yat-sen University were enrolled from January 2021 to March 2025. The general information were collected based on medical records, including patients’ name, gender, age, affected side, affected nerves, duration, etiologic diagnoses, medical histories, the number of PRP injections received, as well as their treatment frequency.

PRP therapy was not administered as a first-line intervention. All enrolled patients had previously received and failed standard conservative treatments for peripheral nerve injuries, including physiotherapy, exercise rehabilitation, corticosteroid injections, and glucose hydrodissection. The duration of prior conservative treatment was at least 3 months in most cases, without satisfactory clinical improvement. Therefore, in this patient population, PRP was used as a later-line, rescue therapy rather than a first-line intervention.

Regarding cohort composition: The study cohort consisted primarily of patients with compressive neuropathies (e.g., carpal tunnel syndrome, cubital tunnel syndrome, common peroneal nerve entrapment), which share similar underlying pathophysiological mechanisms. A small number of patients had other etiologies (toxic, ischemic, or traumatic nerve injury without complete transection). Given the limited sample size—reflecting that PRP is expensive and few patients receive it for peripheral nerve injuries—we pooled all eligible cases to achieve sufficient statistical power for exploratory analyses. The primary objective was to explore the potential applicability of PRP across different peripheral nerve disorders, not to compare efficacy across etiologies. The etiology classification of peripheral nerve injury cases treated with PRP is shown in Table 1.

**TABLE 1:** The etiology classification of peripheral nerve injury cases treated with PRP.

Etiology	Cases (n)	Proportion (%)
Compressive injury	30	62.5
Traumatic injury	11	22.9
Latrogenic injury	2	4.2
Inflammatory injury	1	2.1
Ischemic injury	1	2.1
Toxic injury	3	6.2

Inclusion criteria encompassed the following: 1) Diagnosis of peripheral nerve injury confirmed by clinical presentation (motor and/or sensory deficits in a peripheral nerve distribution), electrodiagnostic studies (nerve conduction studies and/or electromyography), and/or neuromuscular ultrasound; 2) Failure to respond to standard conservative treatments (including physiotherapy, exercise rehabilitation, corticosteroid injections, and/or glucose hydrodissection) prior to PRP injection; 3) Received ultrasound-guided PRP injection for the diagnosed peripheral nerve injury; 4) Age ≥18 years; 5) Ability to comply with the clinical follow-up requirements (1, 3, and 6 months post-injection).

Exclusion criteria: 1) The injured nerve underwent surgical treatment (e.g., functional reconstruction surgery) during the follow-up period; 2) Presence of a malignant tumor or other life-threatening condition that precluded completion of the 6-month follow-up; 3) Lost to follow-up (missing ≥2 consecutive visits).

### Preparation of PRP

PRP was prepared using a double-centrifugation method with a commercial kit (TriCell PRP preparation kit, Rev-Med Inc., Korea). Approximately 30 mL of venous blood was drawn from each patient into sterile tubes containing anticoagulant (citrate dextrose). The blood was first centrifuged at 3,000 rpm for 4 min to separate red blood cells from plasma. The supernatant (plasma and buffy coat) was then centrifuged at 3,300 rpm for 3 min to concentrate platelets. The final pellet was resuspended in a small volume of plasma, yielding approximately 3.5 mL of PRP per patient.

The PRP used in this study was leukocyte-rich PRP (LR-PRP). Based on previously published data characterizing the TriCell preparation system, this method consistently achieves an approximately 3.0-fold enrichment of platelets over whole blood baseline, with significantly elevated leukocyte concentrations (10.09 ± 4.29 × 10^3^/mL, predominantly lymphocytes and monocytes) compared with whole blood (approximately 5.09 × 10^3^/mL) [13]. Quality control tests performed in our laboratory confirmed the reproducibility of this preparation method, consistently achieving a 3- to 5-fold platelet enrichment corresponding to a final platelet concentration of approximately 1,000 × 10^9^/L (1,000,000 platelets/μL), with leukocyte concentrations significantly above whole blood baseline.

However, due to the retrospective nature of this study, platelet and leukocyte counts were not systematically recorded for individual patients at the time of each injection, and we were unable to report exact cell concentrations for the full cohort. To ensure the consistency of our preparation method throughout the study period, we implemented a quality control protocol, performing validation assays (including platelet and leukocyte counts) on a monthly basis. These tests confirmed the reproducibility of the preparation method across different batches.

All PRP injections were performed under ultrasound guidance. The volume injected per nerve depended on the size and location of the affected nerve (the PRP preparation yielded approximately 3.5 mL per patient, which was typically sufficient for one nerve; for patients with bilateral or multiple nerve involvement, a single PRP preparation was sometimes divided between nerves).

### Injection method of PRP

The ultrasound-guided injection technique for PRP varied depending on the diverse anatomic locations of the peripheral nerves, consistent with the injection method outlined in previous research [14]. In summary, the procedures involved the following steps: Firstly, the lesion site of the nerve to be injected was precisely pinpointed through ultrasound examination by an experienced rehabilitation physician. Secondly, an appropriate puncture route is chosen, and the puncture area is thoroughly disinfected to ensure sterility. Thirdly, the puncture is precisely conducted under real-time ultrasound guidance, as demonstrated in Figure 1. Once the puncture needle is positioned adjacent to the target nerve, PRP is gently injected,to make sure that the nerve was surrounded by PRP liquid. Lastly, following the injection, patients are observed in the injection room for a minimum of 10 min to monitor any immediate reactions or complications.

**FIGURE 1:**
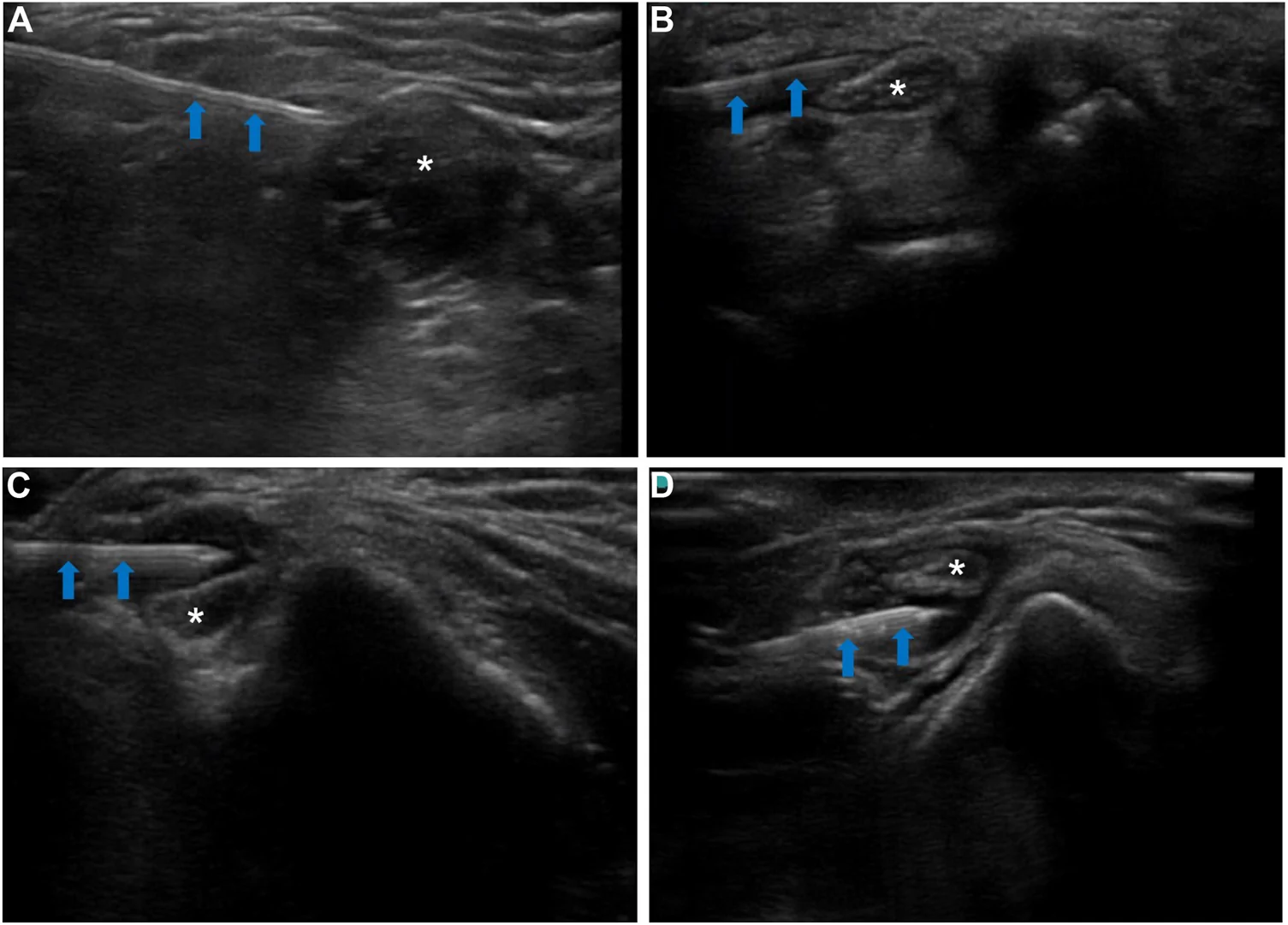
PRP injection for common peripheral nerve injury under ultrasound guidance. Arrow directed towards: the puncture needle. Asterisk denotes: the target nerve. **(A)** Brachial plexus nerve; **(B)** Median nerve; **(C)** Ulnar nerve; **(D)** Common peroneal nerve.

The number of PRP injections and the intervals between them were determined based on the patient’s clinical response, with a generally recommended interval of 1–2 weeks. The majority of patients (approximately 75%) received 2 to 4 injections at 1- to 2-week intervals. Deviations from this schedule occurred in some cases: a few patients received only a single injection, primarily due to financial constraints, transportation difficulties, or unrealistic expectations regarding the speed of therapeutic response—despite being advised that clinical improvement may require time to manifest. Conversely, one patient received up to 8 injections, as each prior injection yielded noticeable improvement, and the patient strongly requested continued treatment.

### Functional assessment

#### Assessment of improvement in predominant sensory symptoms

The Numerical Rating Scale (NRS) was used to assess improvement in patients’ predominant subjective sensory abnormalities. Patients rated their primary sensory symptom on an 11-point scale from 0 (no symptom or normal sensation) to 10 (worst possible symptom). The outcome was defined as subjective improvement in the primary sensory symptom, rather than “sensory recovery,” to more accurately reflect what was measured.

The definition of sensory improvement varied according to each patient’s predominant complaint:

For patients whose primary complaint was pain (e.g., neuropathic pain), sensory improvement was defined as a reduction of ≥2 points on the NRS pain score.

For patients whose primary complaint was numbness, paresthesia, or other abnormal sensations (e.g., formication, burning, or reduced sensation), sensory improvement was defined as a reduction of ≥2 points on the NRS score for that specific symptom.

This approach was adopted because our patient population was heterogeneous in terms of their primary sensory symptoms. The ≥2-point threshold was chosen based on previous studies suggesting that a 2-point reduction represents a clinically meaningful change on the NRS [15].

#### Motor function assessment

The Medical Research Council (MRC) Muscle Strength Grading Scale was used to assess improvement in motor function [16], specifically targeting key muscles innervated by the affected peripheral nerve(s). Per the MRC criteria: Grade 0 indicates no contraction; Grade 1, trace contraction without movement; Grade 2, full range of motion (ROM) with gravity eliminated; Grade 3, full ROM against gravity; Grade 4, full ROM against moderate resistance; Grade 5, full ROM against maximum resistance (normal strength). While achieving a muscle grade of ≥3 is often considered clinically significant as it enables functional movement against gravity, in this study, a ≥1-grade improvement in MRC score from baseline for the key muscles was defined as an effective improvement in motor function. Specifically, the following changes were considered improvement: grade 0 to 1, 1 to 2, 2 to 3, 3 to 4, or 4 to 5. No change (e.g., grade 1 to 1) or any decrease in grade was not considered improvement.

This definition was chosen because some patients had baseline MRC grades above 3 (e.g., grade 4). Defining improvement solely as “achieving MRC grade ≥3” would not capture meaningful improvement in these patients, as they had already surpassed this threshold. Conversely, defining improvement solely as “achieving grade 3” would also fail to detect clinically meaningful improvements in patients who started below grade 3 but did not fully reach grade 3 (e.g., improvement from grade 1 to grade 2). Therefore, we believe the ≥1-grade increase definition is more inclusive and appropriate for our heterogeneous cohort.

#### Assessment time points

Evaluation time points were established as follows: baseline assessment was conducted prior to the first injection of PRP; subsequent evaluations were scheduled at 1 month, 3 months, and 6 months after the initial PRP injection. These time points allowed for a comprehensive assessment of the patient’s progress and response to treatment over time.

### Evaluation of adverse effect

Safety was assessed at each follow-up visit (1, 3, and 6 months post-injection). Using a structured interview format, all patients were systematically asked about the following potential adverse events: Pain at the injection site; Swelling, redness, or warmth; New or worsening numbness or paresthesia; New or worsening weakness; Any other new symptoms since the previous visit.

All adverse events were recorded and categorized by type, severity, and duration.

### Data analysis

Continuous variables are presented as mean ± standard deviation (SD) if normally distributed or median with interquartile range (IQR) if non-normally distributed. Categorical data are expressed as proportions (%).

No *a priori* sample size calculation was performed, as this was a retrospective, exploratory study. The sample size was determined by the number of eligible patients treated during the study period.

Patients were stratified by symptom duration: <1 year vs. ≥1 year. Improvement rates (proportions achieving clinically meaningful sensory and motor improvement) were compared between duration subgroups at 1, 3, and 6 months post-injection using chi-square or Fisher’s exact tests. We performed 3 comparisons (at 1, 3, and 6 months) for each outcome. To address the risk of type I error due to multiple testing, we applied a post-hoc Bonferroni correction. With 3 comparisons, the corrected significance threshold was α = 0.05/3 = 0.0167. After applying this correction, none of the comparisons remained statistically significant. Therefore, our findings should be considered hypothesis-generating and exploratory rather than confirmatory.

To address the concern that a ≥1-grade improvement in MRC score may overestimate clinical benefit in patients with mild baseline deficits, we performed a sensitivity analysis restricted to patients with baseline MRC <3 (n = 16). In this analysis, motor improvement was redefined using a stricter criterion: achievement of MRC grade ≥3 (movement against gravity).

## Results

### General information of the patient

A total of 37 patients were included in this study, of which 11 patients presented with bilateral nerve injuries, resulting in a total of 48 cases receiving injections on the affected side. The general information of the patient undergoing PRP therapy is presented in Table 2.

**TABLE 2:** General information of the patient undergoing PRP therapy.

Characteristic	Category	Value
Age, years	​	42.7 ± 14.5
Sex	Male	21 (56.8%)
​	Female	16 (43.2%)
Affected side	Unilateral	26 (70.3%)
​	Bilateral	11 (29.7%)
Disease duration	<12 months	25 (52.1%)
​	≥12 months	23 (47.9%)
Baseline NRS score	​	6 (IQR: 5-7)
Baseline muscle strength	<grade 3	16 (33.3%)
​	≥grade 3, <grade 5	20 (41.7%)
​	Grade 5	12 (25.0%)

Table 3 outlines the distributions of injured peripheral nerves that undergo PRP treatment. Notably, median nerve injury was the most commonly treated peripheral nerve with PRP. Occasionally, more than two nerves were injected simultaneously.

**TABLE 3:** The distribution of injured peripheral nerves treated with PRP.

Injured nerve site	Cases (n)	Proportion (%)
Brachial plexus nerve and its branches	6	12.5
Median nerve	20	41.7
Ulnar nerve	3	6.2
Radial nerve	5	10.4
Axillary nerve	1	2.1
Sciatic nerve and its branches	7	14.6
Posterior tibial nerve	1	2.1
Common peroneal nerve	5	10.4

### Efficacy analysis

A month after PRP therapy, sensory function enhancement peaked, while motor function improvement was most evident 3 months later, as shown in Table 4. It is noteworthy that the motor function of 13 nerves was normal at baseline.

**TABLE 4:** Comparison of sensory and motor functional recovery rates following PRP treatment at 1-, 3-, and 6-month follow-ups*.

Follow-up	Functional improvement	Cases (n/N)	Rate (%)
1 month	Sensory function	34/48	70.8%
​	Motor function	18/36	50.0%
3 months	Sensory function	32/48	66.7%
​	Motor function	24/36	66.7%
6 months	Sensory function	33/48	68.8%
​	Motor function	25/36	69.4%

* All participants completed 1-, 3-month and 6-monthe valuations. Motor analysis was restricted to 36 cases with baseline MRC, grade <5.

### Sensitivity analysis for non-independence of observations

To address the concern that bilateral cases from the same patient may violate the assumption of independent observations, we performed a patient-level sensitivity analysis including only the more severely affected nerve for patients with bilateral involvement. The results were compared with the original nerve-level analysis.

As shown in Table 5, the responder rates were similar between the two analyses across all time points for both sensory and motor outcomes. Cohen’s h effect sizes ranged from 0.02 to 0.14, all well below the threshold for a small effect (0.2), indicating high consistency. This sensitivity analysis confirms that our main conclusions are robust and not substantially influenced by the inclusion of bilateral cases.

**TABLE 5:** Sensitivity analysis for non-independence of observations*.

Outcome	Time point	Analysis	N	Responder rate (%)	Cohen’s h
Sensory	1 month	Original	48	70.8% (34/48)	0.11
​	​	Sensitivity	37	75.7% (28/37)	​
​	3 months	Original	48	66.7% (32/48)	0.14
​	​	Sensitivity	37	73.0% (27/37)	​
​	6 months	Original	48	68.8% (33/48)	0.09
​	​	Sensitivity	37	73.0% (27/37)	​
Motor	1 month	Original	36	50.0% (18/36)	0.03
​	​	Sensitivity	31	48.4% (15/31)	​
​	3 months	Original	36	66.7% (24/36)	0.02
​	​	Sensitivity	31	67.7% (21/31)	​
​	6 months	Original	36	69.4% (25/36)	0.03
​	​	Sensitivity	31	71.0% (22/31)	​

* Cohen’s h compares original vs. sensitivity analysis; h < 0.2 indicates no meaningful difference.

### Sensitivity analysis for motor improvement definition in patients with baseline MRC <3

Among the 16 patients with baseline MRC <3, we compared the primary definition (≥1-grade increase) with a stricter definition (achieving MRC grade ≥3). The results are shown in Table 6. At 6 months, the improvement rate was 75.0% (12/16) under the primary definition and 68.8% (11/16) under the stricter definition, with an absolute difference of 6.2%. This indicates that most patients who achieved a ≥1-grade improvement by 6 months also reached the clinically meaningful threshold of MRC ≥3.

**TABLE 6:** Sensitivity analysis for motor improvement definition in patients with baseline MRC <3 (n = 16).

Time point	Primary definition (≥1-grade increase)	Stricter definition (achieving MRC ≥3)	Absolute difference
1 month	43.8% (7/16)	25.0% (4/16)	−18.8%
3 months	68.8% (11/16)	50.0% (8/16)	−18.8%
6 months	75.0% (12/16)	68.8% (11/16)	−6.2%

In an exploratory subgroup analysis, patients were divided into Group A (symptom duration <1 year, n = 25 for sensory, n = 20 for motor) and Group B (symptom duration ≥1 year, n = 23 for sensory, n = 16 for motor), as shown in Table 7.

**TABLE 7:** Sensory and motor improvement rates stratified by disease duration (<1 vs. ≥ 1 year)*.

Evaluation	Time	Group A (<1 year)	Group B (≥1 year)	χ^2^	p-value
Sensory improvement rate	1 month	80.0% (20/25)	60.9% (14/23)	2.122	0.145
​	3 months	76.0% (19/25)	56.5% (13/23)	2.045	0.153
​	6 months	80.0% (20/25)	56.5% (13/23)	3.074	0.080
Motor improvement rate	1 month	55.0% (11/20)	43.8% (7/16)	​	0.738
​	3 months	80.0% (16/20)	50.0% (8/16)	​	0.081
​	6 months	85.0% (17/20)	50.0% (8/16)	​	0.034

* Motor analysis was restricted to 36 cases with baseline MRC, grade <5.

For sensory improvement, the responder rate at 6 months was 80.0% in Group A versus 56.5% in Group B (p = 0.08), which did not reach statistical significance but suggested a trend toward better outcomes in patients with shorter symptom duration.

For motor improvement, the responder rate at 6 months was 85.0% in Group A versus 50.0% in Group B (p = 0.034), as shown in Table 7. However, after applying Bonferroni correction for multiple comparisons (3 comparisons: 1, 3, and 6 months; corrected α = 0.05/3 = 0.0167), this p-value no longer remained statistically significant. Given the post-hoc nature of this analysis and the limited sample size, this finding should be interpreted as hypothesis-generating and requires validation in future prospective studies with pre-specified subgroup hypotheses.

For the subgroup analysis comparing patients with symptom duration <1 year versus ≥1 year, we performed 3 independent comparisons (at 1, 3, and 6 months). The same Bonferroni correction (α = 0.0167) was applied. The observed p-value of 0.034 for motor improvement at 6 months did not remain significant after correction. This subgroup analysis was post-hoc and underpowered; the findings should be interpreted as preliminary observations requiring validation in future studies.

### Analysis of adverse effect

A total of 37 patients (48 nerves) received perineural PRP injections. One adverse event was observed, occurring in 2.7% of patients (1/37) and 2.1% of nerves (1/48).

Description of the event: In this case, the needle tip inadvertently penetrated the epineurium, resulting in partial intraneural injection of PRP. The patient experienced transient dysesthesia in the affected nerve distribution, which resolved completely within 2 months without any specific intervention.

No other adverse events were reported. Specifically, there were no cases of infection, hematoma, persistent pain, permanent neurological worsening, or allergic reactions. For all injections in which the needle tip was confirmed to be positioned outside the epineurium, no adverse events occurred.

## Discussion

This study revealed that ultrasound-guided PRP therapy for peripheral nerve injury may be associated with the recovery of both sensory and motor functions during a 6-month follow-up period and appeared safe. However, given the lack of a control group and the retrospective design, these findings should be considered preliminary and hypothesis-generating.

Currently, there is a scarcity of reports detailing the clinical application of PRP in the treatment of peripheral nerve injury. Several studies have shown that PRP could be employed in the management of peripheral nerve compression injuries, particularly in cases like carpal tunnel syndrome [17, 18]. Additionally, a single case report demonstrates the successful application of PRP in treating traumatic peripheral nerve injury, specifically involving the radial nerve [19]. Furthermore, clinical studies to investigate the effectiveness of PRP in treating diabetic peripheral neuropathy [20] and leprosy peripheral neuropathy [21], have indicated its potential in diverse neuropathic conditions. In this study, we have successfully employed PRP in treating peripheral nerve injuries stemming from diverse etiological factors, and the encouraging outcomes underscore the promising application of PRP in managing peripheral nerve injuries of various causes.

The safety profile of perineural PRP injection in this study was favorable. Only one adverse event (transient dysesthesia due to inadvertent intraneural injection) occurred, which resolved completely within 2 months. This highlights the importance of precise needle placement under ultrasound guidance to avoid intraneural injection. These findings suggest that perineural PRP injection is generally safe when performed with careful technique. However, given the small sample size, rare adverse events cannot be excluded. Larger prospective studies are needed to better define the safety profile of this procedure.

In this study, none of the patients reported experiencing serious adverse effect during the injection process or during the follow-up period, suggesting that PRP is a safe therapeutic option for peripheral nerve injury, due to its autologous origin derived from the patient’s own blood [22], which is consistent with previous reports in the literature [23]. In addition to its safety and reliability, both clinical studies and animal experiments have indicated that PRP can facilitate the repair of injured nerves [8, 24]. Malahias’ study yielded profound insights, revealing that a single injection of PRP led to a substantial improvement in VAS outcomes, coupled with a marked enhancement in upper limb functionality among CTS patients within a month [25]. In Hakan’s study on CTS, it was observed that after a 3-month period, the PRP group demonstrated significantly superior Boston Carpal Tunnel Questionnaire scores in terms of both symptom severity and functional capacity, in comparison to the corticosteroid group [17]. As previously reported, a patient suffering from common peroneal nerve palsy for 11 months demonstrated remarkable improvements following PRP treatment, and complete reinnervation of the peroneus longus muscle and enhanced reinnervation of the tibialis anterior muscle at 21 months after the first injection [26]. These findings offered an alternative treatment for patients with acute and chronic peripheral nerve injuries.

Regarding the subgroup analysis based on symptom duration: At the 6-month follow-up, patients with shorter symptom duration (<1 year) showed numerically higher motor improvement rates compared to those with longer duration (85.0% vs. 50.0%, p = 0.034). A similar trend was observed for sensory improvement (80.0% vs. 56.5%, p = 0.08). However, after applying Bonferroni correction for multiple comparisons (corrected α = 0.0167), the motor improvement did not remain statistically significant. Therefore, these findings should be interpreted with caution and considered hypothesis-generating rather than definitive evidence of a differential treatment effect. This temporal disparity likely stems from two factors: 1) PRP’s synergistic action with preserved regenerative mechanisms in acute/subacute injuries, and 2) the progressive deterioration of endogenous repair capacity in chronic cases. Mechanistically, PRP may enhance early repair by leveraging residual Schwann cell activity and a permissive microenvironment [11, 27]. Thus, PRP’s therapeutic window may be most favorable in acute/subacute injuries, where its effects complement residual repair capacity. In chronic cases, its efficacy may be limited by both Schwann cell senescence and a fibrosis-dominated microenvironment [27, 28].

Our study revealed a distinct temporal pattern of functional recovery. Sensory improvements occurred earlier, with recovery rates of 70.8%, 66.7%, and 68.8% at 1, 3, and 6 months post-injection, respectively. In contrast, motor recovery progressed more gradually, with improvement rates of 50.0%, 66.7%, and 69.4% at the corresponding time points. This finding aligns with Wu et al.’s observations in carpal tunnel syndrome patients, where PRP provided rapid pain relief (evident by 1 month) but required 6 months for full functional recovery [23]. The differential recovery rates suggest that PRP may promote early sensory restoration, whereas motor recovery follows a more prolonged timeline. This biphasic pattern—early sensory enhancement followed by delayed motor improvement—has been less frequently reported in PRP-related studies. We hypothesize that this temporal disparity may reflect PRP’s ability to rapidly modulate inflammatory processes, thereby alleviating symptoms more quickly [29], while axonal regeneration and neuromuscular reinnervation, being challenging processes, progress more slowly, as documented by Fuentes-Flores et al [30]. However, further experimental validation is needed to elucidate the underlying mechanisms.

However, it is important to note that when PRP treatment fails to produce satisfactory results, several crucial considerations arise. Firstly, it is crucial to ascertain whether the underlying causes of peripheral nerve injuries have been clearly identified. Secondly, it is essential to ensure that the specific lesion sites targeted by PRP therapy are accurately defined. Using ultrasound guidance, we treated a wide range of peripheral nerves, including the brachial plexus, median nerve, ulnar nerve, radial nerve, sciatic nerve, and its various branches. In this study, PRP was most frequently utilized in patients with median nerve injuries, primarily due to the high incidence of upper limb peripheral nerve injuries [4]. With the aid of ultrasound imaging, we can precisely inject PRP into the targeted sites, potentially enhancing therapeutic efficacy and improving patient outcomes.

There is a scarcity of studies examining the frequency of PRP injections. Specifically, in the treatment of CTS, some researchers typically administer a single PRP injection [18, 25]. In the specific case of peroneal nerve injury, one researcher initiated a series of three injections, administered at 2-week intervals, followed by a monthly injection as part of the treatment protocol [26]. In this study, the number of PRP injections ranged from 1 to 8, with a time interval of more than 1 week between injections, and most injections were administered at 2-week intervals. Currently, the clinical use of PRP for treating peripheral nerve injuries is gaining traction. However, there is still no unified standard regarding the frequency and number of PRP treatments for peripheral nerve injuries. In this study, therapeutic strategies were primarily determined based on the patient’s response to treatment, along with other considerations such as the patient’s economic situation.

The main limitation of this study pertains to the motor function assessment criteria. We defined motor improvement as an increase of ≥1 MRC grade, irrespective of the baseline severity. In contrast, previous studies on peripheral nerve injury have focused on patients with severe motor deficits (baseline MRC grade <3) and defined meaningful recovery as achieving a grade of ≥3. This methodological difference may lead to an overestimation of the therapeutic benefit when compared against more stringent benchmarks. Additionally, the retrospective design, small sample size, and lack of a control group constrain the generalizability of our findings. Future investigations should adopt rigorous prospective designs with standardized, clinically relevant outcome measures to further validate the efficacy of PRP in peripheral nerve regeneration.

The present study evaluated outcomes up to 6 months post-treatment. However, peripheral nerve regeneration, particularly after proximal or severe injuries, may require 12 months or longer to become fully manifest. Therefore, the durability of the observed improvements beyond 6 months remains unknown. We are currently extending follow-up for this patient cohort and plan to report 12- and 24-month outcomes in future studies.

### Limitations

Several limitations should be acknowledged. This was a retrospective, single-arm study without a control group, which limits causal inference and may overestimate efficacy. The number and intervals of PRP injections varied in real-world practice, and a dose-response relationship cannot be excluded. Our outcome measures also have inherent limitations: MRC improvement defined as a ≥1-grade increase may overestimate benefit in patients with mild baseline deficits, and patient-reported NRS scores cannot fully distinguish analgesic effects from true sensory restoration. In addition, pre- and post-treatment electrodiagnostic data were unavailable for most patients, limiting objective assessment of nerve regeneration.

Given these limitations, our findings should be interpreted as preliminary and hypothesis-generating. Prospective, randomized controlled trials are needed to confirm the efficacy of PRP for peripheral nerve injuries.

## Conclusion

This retrospective, exploratory study provides preliminary and hypothesis-generating evidence that perineural PRP injection may be associated with functional improvement in selected patients with peripheral nerve injuries, and appears safe. The observed trend toward better outcomes in patients with shorter symptom duration did not withstand Bonferroni correction and requires further validation. Prospective, randomized controlled trials are needed to confirm the efficacy of PRP for this indication.

## Data Availability

The datasets generated and analyzed in this study aren't publicly available because of participants’ privacy concerns, but can be provided by the corresponding author upon reasonable request.
